# Plants Used in Antivenom Therapy in Rural Kenya: Ethnobotany and Future Perspectives

**DOI:** 10.1155/2020/1828521

**Published:** 2020-06-16

**Authors:** Timothy Omara

**Affiliations:** ^1^Department of Chemistry and Biochemistry, School of Sciences and Aerospace Studies, Moi University, Eldoret, Kenya; ^2^Africa Centre of Excellence II in Phytochemicals, Textiles and Renewable Energy, Moi University, Eldoret, Kenya; ^3^Department of Quality Control and Quality Assurance, Product Development Directory, AgroWays Uganda Limited, Jinja, Uganda

## Abstract

Snake envenomation is one of the neglected tropical diseases which has left an intolerable death toll and severe socioeconomic losses in Kenya. In a continued effort to identify some antiophidic East African botanical species, this study generated ethnobotanical information on antivenom plants reported in Kenya, with a view to identify potential species which could be subjected to *in vitro* and clinical studies for possible development into antivenoms. Data retrieved through searches done in multidisciplinary databases (Scopus, Web of Science, PubMed, Science Direct, Google Scholar, and Scientific Electronic Library Online) indicated that 54 plant species belonging to 45 genera, distributed among 27 families, are used for the management of snakebites in Kenya. Most species belonged to the family Asteraceae (11%), Malvaceae (11%), Fabaceae (9%), Annonaceae (6%), Combretaceae (6%), and Lamiaceae (6%). The main growth habit of the species is as herbs (35%), shrubs (33%), and trees (28%). Ethnomedicinal preparations used in treating snake poisons are usually from leaves (48%), roots (26%), and stem bark (8%) through decoctions, infusions, powders, and juices which are applied topically or administered orally. The most frequently encountered species were *Combretum collinum*, *Euclea divinorum, Fuerstia africana*, *Grewia fallax*, *Microglossa pyrifolia*, *Solanecio mannii*, and *Solanum incanum*. Indigenous knowledge on medicinal antivenom therapy in Kenya is humongous, and therefore studies to isolate and evaluate the antivenom compounds in the claimed plants are required to enable their confident use in antivenom therapy alongside commercial antivenin sera.

## 1. Introduction

Envenomation by snakes is one of the major causes of deaths recorded worldwide and has been flagged by the World Health Organization as a neglected tropical disease [1]. Globally, more than 5.5 million snake envenomations are encountered yearly, 2 million victims of which succumb to death while 0.4 million acquire permanent disability [1, 2]. Up to 500, 000 of these cases are reported annually in Africa [3–5]. Of these, more than 70,000 cases are from East Africa, and the situation has been exacerbated by climate change and deforestation which push snakes out of their natural habitats into the general population [6]. In 1971, 1972, and 1973, there were 89, 67, and 22 snakebite deaths recorded in Kenya [7]. In 1994, 151 snakebite cases were reported, with 19% being from venomous snakes [8]. Between 2007 and 2016, snakes injured 7,772 Kenyans, killing 614. Snakebite cases in the Baringo county alone in this period ranged between 300 and 500 cases per month [9, 10]. The cases are typically high at the start of rainy seasons, when snakes come out of their shelter to hunt and breed [11]. Thus, 15 to 25 Kenyans die daily due to snake envenomation, and more than 100 others have their limbs amputated, causing them permanent disability [11]. Most bites are attributed to circumstantial stepping on the snakes by unprotected or barefooted victims [12] and snake ecology [13] while others are instituted by malevolent or drunk victims [6, 14].

East Africa is a home to about 200 species of snakes of the over 400 species found in Africa. The puff adder (*Bitis arietans*), Gabon viper (*Bitis gabonica*), green or Jameson's mamba (*Dendroaspis jamesoni*), black mamba (*Dendroaspis polylepis*), forest cobra (*Naja melanoleuca*), and black-necked spitting cobra (*Naja naja nigricollis*) are the most venomous serpents reported in Kenya [15, 16]. Puff adders are the main envenoming serpents in Kenya for the fact that they are nocturnal and naturally well camouflaged. Their venoms are potently cytotoxic, causing severe pain, swelling, and blistering and in many cases are accompanied by severe tissue damage [6]. The black-necked spitting cobra is another common species which usually spits its venom into the eyes of the victims [6].

In its move to revise the Wildlife Conservation and Management Act 2013, the Kenya Wildlife Service noted that compensation claims for venomous snakebites accounted for 81% of all human-wildlife conflict claims [17]. These claims accumulated to billions of Kenya shillings over the years, prodding the Tourism and Wildlife ministry to declare the situation a crisis that threatens a bankrupt country. The burden led to the ousting of snakebites from the zoological list of compensable human-wildlife conflict claims [6, 17]. The hot spots in Kenya with serious reports of snake envenomation include Baringo, Tharaka-Nithi, Taita Taveta, Kilifi, Kitui, Wajir, Garissa, Machakos, Marsabit, Isiolo, and Makueni [11, 16–19].

Antivenom sera is the only credited treatment for the management of snake envenomation [14, 20–22]. Unfortunately, they are associated with various side effects and may only be effective if administered within 30 minutes to 6 hours of the snakebite. Thus, the barriers to solving the menace of snakebites in Kenya range from poor road networks, fragmented records, and lack of public health education to the absence of antivenom and poor antivenom preservation facilities in health centres [9, 16, 19, 23]. Lack of antivenom sera has been reported globally, and several attempts have been made to develop snake venom antagonists from other sources [24, 25] to supplement those derived from horses. In the East African case, some studies indicated that some antivenom sera on the market were ineffective against some medically important snake venoms [15, 26]. Thus, up to 85% of Kenyan snakebite victims seek treatment from traditional medicine practitioners or use rudimentary means to eliminate the venom [17, 27]. The current study gives an overview of traditional management of snake envenomation in Kenya, utilizing medicinal plants.

## 2. Methodology

Relevant literature pertaining to snakes, snakebites, and their management using medicinal plants in Kenya were sourced from Scopus, Web of Science Core Collection, PubMed, Science Direct, Google Scholar, and Scientific Electronic Library Online from November 2019 to January 2020, following procedures used in previous studies [28, 29]. The main search words used were snakebite, snake envenomation, vegetal, traditional medicine, ethnobotany, alternative medicine, ethnopharmacology, antivenom, antivenin, antiophidian, antitoxin, antidote, and Kenya. The search was performed independently in all the databases. The study databases included original articles published in peer-reviewed journals, books, thesis, dissertations, patents, and other reports covering antivenin plants, dated until January 2020. All publishing years were considered in this narrative review, and articles with information on snakes, snakebites, or medicinal plants in Kenya were given the utmost priority.

Thus, references contained within the returned results were assessed concerning their inclusion in this study, and further searches were carried out at the Google search engine using more general search terms, to broaden the search as follows: words such as snake, plants, plant extract, vegetal, vegetal species, vegetal extract, traditional medicine, alternative medicine, complementary therapy, natural medicine, ethnopharmacology, ethnobotany, herbal medicine, herb, herbs, decoction, infusion, macerate, envenomation, *nyoka (*Swahili word for snake), snakebite, venom, antivenin, antivenom, and Kenya. The last search was done on 20 January 2020. The search outputs were saved where possible on databases, and the author received notification of any new searches meeting the search criteria from Science Direct, Scopus, and Google Scholar.

The scientific names of the plants listed were vetted in botanical databases: the Plant List [30], International Plant Names Index [31], NCBI taxonomy browser [32], and Tropicos [33], where a given species was considered as a distinct species in different reports, and the nomenclature as per the botanical databases took precedence. The families, local names (Luo, Kamba, Kikuyu, Markweta, Massai, and Swahili), growth habit, part (s) used, preparation, and administration mode were captured.

## 3. Results and Discussion

Only articles in English with information on antivenom plants or therapy were considered.

### 3.1. Perceptions about Snakes, Snakebites, and Antivenin Plants Used in Rural Kenya

From the information retrieved, it is clear that the local communities in Kenya are aware of snakebites and have different perceptions about snakes. The beliefs appear to be tribe related, and include snakes “can protect and are signs of fortune” among the Luo or “are connected to witchcraft” in most communities [11, 34–36]. The Luo for example believes that the sacred snake (*Omieri*) which lives in Lake Victoria (Nyanza) often surface in times of drought to “summon” the rains [35]. Among the Samia, the serpents are treated as gods, and some residents pray to them for prosperity of their families and businesses [36].

Most Kenyans know that their current social conditions such as chronic poverty, sleeping in thatched and brick-walled houses, and occupation such as cultivation, hunting, and herding cattle increase the chances of getting bitten by a snake [12, 14, 18]. Snakebites are always treated as exigencies, and the economic implications are perceived to be high due to the cost of transport to traditional healers or health facilities, the care needed by the victims, enforced borrowing, loss of legs, arms, income, and time, and sometimes death [14, 16].

**Figure 1:**
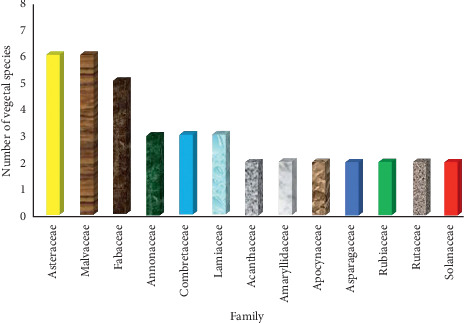
Major families from which vegetal antivenoms are obtained in rural Kenya.

This study retrieved 54 plant species from 45 genera belonging to 27 botanical families used in antivenin therapy in Kenya (Table 1). The common families were Asteraceae (11%), Malvaceae (11%), Fabaceae (9%), Annonaceae (6%), Combretaceae (6%), and Lamiaceae (6%) (Figure 1).

Most families encountered have been reported to have a potential for treating or avoiding snakebites in other countries. Apocynaceae, Asteraceae, Convolvulaceae, Fabaceae, and Myricaceae were cited in Uganda, Tanzania, Pakistan, Ethiopia, Djibouti, and Nigeria [28, 51–55]. Acanthaceae, Apocynaceae, Asteraceae, Euphorbiaceae, Fabaceae, Rubiaceae, and Rutaceae were reported in Bangladesh [56, 57], Central America [58], and India [59].

### 3.2. Growth Habit, Parts Used, Preparation, and Administration Methods of Antivenin Plants Used in Rural Kenya

**Figure 2:**
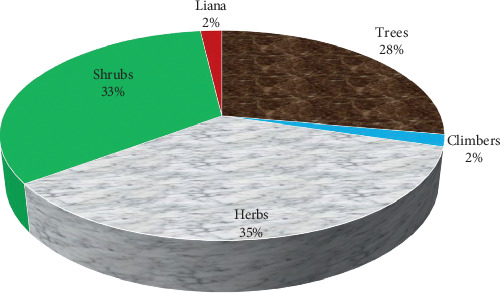
Life forms of the antivenom plants used in rural communities of Kenya.

**Figure 3:**
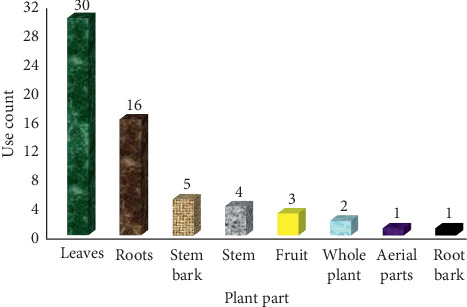
Different parts of antivenom plants used for management of snakebites in Kenya.

The use of plants for treatment of snakebites is not novel in rural communities, and cases handled using antivenin plants rarely record victims succumbing to death [57, 60, 61]. Antivenom plants used in Kenya are majorly herbs (35%), shrubs (33%), and trees (28%), and the commonly used plant parts are leaves (48%), roots (26%), and stem bark (8%) (Figures 2 and 3). The regular use of roots and leaves in antivenom preparations is a characteristic feature of traditional antivenom therapy [40, 52, 62, 63], and no wonder some of these plants are named “snakeroot” in some rural communities [64]. Comparatively, embryonal plant parts such as fruits, seeds, buds, bulbs, and flowers which have reputation for accumulating bioactive compounds are less frequently utilized, concordant with the reports from other countries [28, 52]. Antivenom preparations are often decoctions, infusions, or poultices applied at the point of snakebite (topically) or ingested orally.

In this study, it was noted that few plant species are used against snakebites simultaneously in different locations. This could probably be attributed to the abundant distribution of the analogue active substances among the vegetal species, especially those from the family Asteraceae and Fabaceae. Some of the plants listed are also used for killing, wading off, or discouraging snakes from reaching human and livestock abodes. In most instances, the plants produce pungent odours which cause discomfort and disorientation to snakes when they slither over them. For example, the leaf sap of *Sansevieria intermedia* (locally known as *Sorogat*) is used as a bait to kill snakes [44]. In addition, maintaining a container of water some distance away from the house has been reported for dissuading snakes [11, 12, 65]. Burning of materials such as a piece of cloth has also been noted to discourage snakes [65], similar to tyre burning reported among the Lango of Uganda by Omara et al. [28].

Nearly, all the plants identified in this review are employed for the treatment of various ailments in Kenya and in other countries. For example, *Bidens pilosa* L. either as a whole plant or the different parts has been reported to be useful in the treatment of more than 40 disorders including inflammation, immunological disorders, digestive disorders, infectious diseases, cancer, metabolic syndrome, and wounds [67, 68]. Such plants tend to be used in different communities for treating snakebites and there can be a justification of their efficacy. On the other hand, some of the antivenom plants cited tend to exhibit toxicity. For example, *Catharanthus roseus* (L.) G. Don has neurotoxic alkaloids, especially vincristine [68]. Vincristine and vinblastine are highly toxic antimitotics that block mitosis in the metaphase after binding to the microtubules [70]. Side effects such as myelosuppression, alopecia, abdominal cramps, constipation, nausea, paralytic ileus, ulcerations of the mouth, hepatocellular damage, kidney impairment, pulmonary fibrosis, urinary retention, amenorrhoea, azoospermia, orthostatic hypotension, and hypertension [71] have been reported for anticancer drugs vincristine and vinblastine derived from this plant. This observation explains in part, why some antivenom preparations in Kenya are applied topically or ingested in small amounts. Fortuitously, topical application is a better approach for reducing the local action of the venoms at the bitten sites [28].

### 3.3. Treatment of Snakebites

Antivenom therapy in rural Kenya usually involves monopreparations of plant extracts, while in a few cases, mixtures of different plant species and parts are used to prepare the antidotes. Some plants are used for treating envenomation by specific snakes. For example, *Conyza sumatrensis* (Retz.) E. Walker leaf infusion is used for management of puff adder bites [14, 34]. *Maesa lanceolata* Forssk. root bark decoction is then administered as a follow-up for such bites. Use of black stone (viper stone) has been cited in some parts of Kenya [9, 12, 36, 72]. The carbonized material (a piece of processed bone from a cow's thigh bone) is placed on the bite where it attaches and “sucks out” the venom. The stone, known as *ekina erienjukha* or *ekina emari* in the local Kisamia dialect, can be used several times for different victims and also alongside other treatment options, provided it is sterilized in boiling water for at least 15 minutes and then soaked in milk for two hours to neutralize it [36].

Among the Luo, oral administration of egg yolk and albumin prior to treatment was recorded [14]. In some communities, mystical therapies have been reported [27]. For instance, the snake “teeth” are crushed and mixed with a powder of burnt *Opilia amentacea* roots and applied to the bite. Other practices such as burning matchstick at the site of the bite, application of vaseline and tourniquets (or clothes impregnated with charcoal), and pouring paraffin and cold water on the bite have been documented [16]. Direct sucking of the venom from the bitten site has been also been cited [73]. Generally, incisions are made around the bite and herbal remedies are applied on the wound site. Antidotes are administered not later than half an hour after the bite and are usually accompanied by solace and advice to the victims [14]. In some instances, traditional healers with spiritual herbal skills may at their discretion invoke spiritual healings [14].

### 3.4. Knowledge Dynamics of Antivenin Therapy in Kenya

Information on antivenom plants and therapy is usually acquired and passed on orally from the elders to the young (apprenticeships from relatives) [14, 50] as is the case with other African countries [28, 74]. Others acquire knowledge through own observations, formal training, or are inducted by mobile herbal practitioners [49]. However, expert healers are either spirit inspired [14] or had ancestral induction through dreams [50].

Most antivenom plants utilized by traditional healers in Kenya have not been fully documented despite the obvious risk of their disappearance owing to threats such as deforestation, overexploitation, and death of traditional healers with knowledge of the plants [14, 75]. This is confounded by the fact that traditional medicine is usually a guarded family secret, and as reported in other African countries [14, 75, 76], children are not always willing to inherit the art because they spend most of their youthful years at school [14, 78]. Most of the antivenom plant species are indigenous (wild and uncultivated), though some such as *Allium cepa*, *Tagetes minuta*, *Senna siamea*, and *Tithonia diversifolia* are naturalized exotic species.

## 4. Future Perspectives

In a view to capture snakebite records, Kenyan health authorities suggested that traditional medicine should be integrated with modern medicine since the former is usually the first point of contact by snakebite victims and is usually the most trusted in their societies [14, 27]. The Kenya Snakebite Research and Intervention Centre predicts to produce East Africa's first antivenom medication within five years at a cost about 30% of the currently imported antisera [79]. Kenya currently extracts venoms from snakes, which are transported to South Africa for manufacturing antivenoms [17]. To reduce the menace of snakebites, the Kenyan government has reached out to train health workers in management of snakebites as well as the local community [80]. It has also keyed in an initiative to establish snake parks notably the Mwingi plant sanctuary in the Kitui county, the Nairobi snake park in Nairobi, the Lake Baringo reptile park, and Dr. Richard Leakey's snake park in the Baringo county. These are expected to reduce the snake population as well as boost tourism industry [81]. Exploration of antivenin plants for their efficacy in snakebite therapy could be one of the interventions in management of snakebites, as interfacing with these serpents are inevitable and treatment of snakebites cannot be managed by commercial antivenins alone.

## 5. Conclusion

The inventory of plants utilized by Kenyan communities present a considerable potential for treatment of snake envenomation and dissuading snakes. More ethnobotanical surveys should be done in the uncovered areas, and health authorities need to devise means of collecting coherent data on snakebites in the country to enhance effective planning for management of the menace.

## Figures and Tables

**Table 1:** Antivenin plants used across Kenyan communities.

Plant family	Botanical name	Local name	Part used	Growth habit	Mode of preparation/administration	Authors
Acanthaceae	*Justicia betonica* L.	Shikuduli	Leaves	Herb	Infusion drunk	[37]
	*Justicia calyculata* (Deflers.) T. Anders	Apiwo, piu piu (Luo)	Aerial parts	Herb	Crushed and rubbed on the bite to facilitate removal of the “snake's fangs”	[14, 34]

Amaryllidaceae	*Allium cepa* L.	Kitunguu (Kamba)	Leaves, roots	Herb	Pounded and sap applied	[14]
	*Ammocharis tinneana (*Kotschy & Peyr.) Milne-Redh	Apap thwon pap, rabwond otenga (Luo)	Roots	Herb	Sap used in preparation of an alexiteric	[14, 34]

Annonaceae	*Annona senegalensis* Pers. sp. *senegalensis*	Obolo, obolobolo (Luo)	Leaves	Tree	Crushed and rubbed on the bite. Some may be chewed, and the juice swallowed	[14, 34]
	*Uvariodendron anisatum* Verdc.	Ndonga	Whole plant, leaves	Shrub	Ashes applied to the bite, also used for scorpion bites	[38, 39]
	*Uvaria scheffleri* Diels	Mukukuma (Kamba)	Root, leaves	Shrub	Dried in the sun, pounded, and powder applied	[14]

Apiaceae	*Steganotaenia araliacea* Hochst.	Muvuavui, kivuavui (Kamba)	Roots	Shrub	Burnt into charcoal, crushed into powder, and applied on the bite	[14, 40]

Apocynaceae	*Carissa spinarum L*.	Mukawa (Kamba)	Leaves	Shrub	Used for treatment and has snake repellent activity	[41]
	*Catharanthus roseus* (L.) G. Don	Olubinu	Leaves	Herb	Infusion drunk	[37]

Asparagaceae	*Sansevieria kirkii* Baker	No local name given	Leaves	Herb	Sap applied on the bite wound	[40]
	*Sansevieria parva* N.E.Br.	Twoch bungu (Luo)	Leaves	Herb	Sap applied on the bite wound	[14, 34]

Asteraceae	*Bidens pilosa* L.	Nyanyiek mon, onyiego (Luo)	Leaves	Herb	Crushed and rubbed on fresh cuts as an astringent, snake bite antidote, and antiseptic	[14, 34]
	*Conyza sumatrensis* (Retz.) E. Walker	Yadh asere, yadh tong' (Luo)	Leaves	Herb	Infusion drunk for puff adder bites	[14, 34]
	*Solanecio mannii* (Hook. f) C. Jeffrey	Maroo, marowo (Luo), livokho	Leaves	Shrub	Crushed or chewed leaves rubbed onto the bite	[14, 34, 37]
	*Tagetes minuta* L.	Muvangi (Kamba)	Leaves	Herb	Crushed, soaked in water, & infusion applied on bite	[14]
	*Tithonia diversifolia* (Hemsl.) A. Gray.	Maua madongo, akech (Luo), mula (Kamba)	Leaves	Shrub	Infusion administered orally	[14, 34]
	*Vernonia glabra* (Steez) Vatke	Olusia (Luo)	Leaves	Herb	Leaf ash or crushed leaves rubbed onto scarifications around the bite	[14, 34]

Bignoniaceae	*Markhamia lutea* (Benth.) K. Schum	Lusiola, shisimbali	Leaves	Tree	Fresh leaf infusion drunk and used for cleaning snake bite wounds	[37]

Burseraceae	*Commiphora africana* (A. Rich.) Endl.	Osilalei	Bark	Tree	Chewed	[42]

Combretaceae	*Combretum collinum* Fresen	Adugo (Luo)	Roots	Tree	For treatments effected by scarification	[14, 34, 40]
	*Combretum molle* G. Don	Muama, kiama (Kamba)	Bark, roots	Tree	Infusion drunk; 2 glasses two times a day	[14]
	*Combretum padoides* Engl. & Diels	No local name given	Leaves	Tree	Crushed leaves are applied on the bite	[43]

Convolvulaceae	*Dichondra repens* J.R. Forst. & G. Forst.	No Luo name given	Leaves	Herb	Rubbed on bite to “remove snake fangs”	[14, 34]

Ebenaceae	*Euclea divinorum* Hiern	Uswet (Markweta)	Bark	Tree	Crushed & applied on incision made on the bite area. Sometimes used with of *Gardenia volkensii* and *Plectranthus barbatus*.	[44–46]

Euphorbiaceae	*Tragia brevipes* Pax.	Kimelei ne mining (Markweta)	Roots	Shrub	Crushed and applied on incisions made on the bitten area	[44, 46]

Fabaceae	*Entada leptostachya* Harms	Mwaitha (Kamba)	Stem	Tree	Stem crushed, sap squeezed out, and applied on the bite	[14]
	*Erythrina excelsa* Baker	Roko, yuoma (Luo)	Bark	Tree	Sap is used as an antidote	[14, 34]
	*Erythrina abyssinica*	Omutembe (Kuria), muhuti (Kikuyu)	Bark	Tree	Sap is used as an antidote	[40]
	*Indigofera circinella* Baker f.	Odolo (Luo)	Leaves	Herb	Poultice chewed and pasted on the bite	[14, 34]
	*Senna siamea* (Lam.) Irwin et Barnaby	Ndege owinu, oyieko (Luo)	Roots	Tree	Used with *Zanthoxylum chalybeum* Engl. roots	[14]

Lamiaceae	*Fuerstia africana* T.C.E. Fr.	Abunga-useke, aremo (Luo)	Leaves	Herb	Crushed and filtered infusion drank	[14, 34, 47]
	*Hyptis pectinata* (L.) Poit	Not specified	Leaves	Herb	Infusion with *Corchorus trilocularis* L. is dropped or sprinkled into the eye to neutralize venom ejected into it by black-necked spitting cobra. After, the victim is scarified on the hind torso. Claims of intense pain, temporary blindness, and watery eyes were linked to this type of envenomation.	[14]
	*Plectranthus barbatus* Andrews	Kan'gurwet (Markweta)	Leaves	Herb	Mixed with those of *Gardenia volkensii* and *Euclea divinorum.* The powder mixture is then applied on incisions made on the bitten area.	[46]

Malvaceae	*Corchorus trilocularis L*.	Apoth (Luo)	Leaves	Herb	Infusion with *Hyptis pectinata* is dropped or sprinkled into the eye to neutralize snake venom ejected into the human eye	[14, 34]
	*Grewia bicolor* Juss.	Esiteti (Massai), mulawa (Kamba)	Roots	Shrub	Infusion drunk	[40, 42]
	*Grewia damine* Gaertn.	Ositeti	Bark, branch, fruits, roots, stem	Shrub	Not specified	[48]
	*Grewia fallax* K. Schum.	Powo (Luo), ilawa (Kamba)	Leaves, bark	Shrub	Used in cooking envenomed carcass to prevent secondary poisoning. Bitten livestock are drenched with a decoction. Mucilaginous crushed leaves used to wipe the bitten area.	[14, 34, 40]
	*Grewia truncate* Mast.	Not given	Leaves	Shrub	Not specified	[40]
	*Triumfetta rhomboidea* Jacq.	Muinda nguue (Kamba)	Roots	Herb	Crushed, aqueous infusion applied on bite area	[14]

Musaceae	*Ensete edule* (J. F. Gmel.) Horan	Kitembe (Luo)	Stem	Herb	Fresh stem sap is wiped onto the bite	[14, 34]

Myricaceae	*Maesa lanceolata* Forssk.	*Katera* (Luo)	Roots	Tree	Decoction administered as follow-up treatment for puff adder bites	[14, 34]

Opiliaceae	*Opilia amentacea* Roxb.	*Mutonga* (Kamba)	Roots	Climber	Roots burnt into charcoal, crushed into powder, mixed with crushed snake teeth, and applied to the bite	[14]

Phytolaccaceae	*Phytolacca dodecandra* L. Hiern	Kupsogotit	Leaves, fruits	Shrub	Burnt, chewed	[45]

Polygalaceae	*Securidaca longepedunculata*		Not specified	Shrub	Not specified	[49]

Pteridaceae	*Pellaea viridis* (Forssk.) Prantl.	No Luo name given	Leaves	Herb	Pulped and rubbed on the bite	[14, 34]

Rhamnaceae	*Ziziphus mucronata* Willd.	Oloilalei	Roots	Shrub	Infusion drunk	[42]

Rubiaceae	*Gardenia volkensii*	Mogilio (Markweta)	Leaves	Tree	Mixed with those of *Plectranthus barbatus* and *Euclea divinorum.* The powder mixture is then applied on incisions made on the bitten area.	[46]
	*Hymenodictyon parvifolium*	Mulinditi	Leaves	Shrub	Crushed and infusion drunk	[50]

Rutaceae	*Toddalia asiatica* (L) Lam.	Mururue (kikuyu)	Root bark	Liana	Infusion	[40]
	*Zanthoxylum chalybeum* Engl.	Oloisuki (massai)	Roots	Tree	Used with the roots of *Senna siamea* (Lam.) Irwin et Barnaby	[14]

Solanaceae	*Solanum incanum* L.	Mutongu (Kamba)	Leaves, stem, fruit	Shrub	Leaves chewed and applied at the bite. Stem/fruits sliced, sun dried, pounded, and powder applied. The sap of the fruits may also be directly applied.	[14, 40, 42]
	*Solanum micranthum* Schltdl.	Sigowet	Root, fruit	Shrub	Treats snake, cat, or dog bite. Burnt or boiled and infusion taken	[45]

Velloziaceae	*Xerophyta spekei* Baker	Kianduri	Whole plant	Shrub	Ashes applied to the bite	[38]

Decoction: water extraction by boiling of plant material; infusion: the plant material is added to hot water, steeped for few minutes, and taken as tea.

## Data Availability

This is a review article, and no raw experimental data were collected. All data generated or analysed during this study are included in this published article.
